# The Methylation Status of the Epigenome: Its Emerging Role in the Regulation of Tumor Angiogenesis and Tumor Growth, and Potential for Drug Targeting

**DOI:** 10.3390/cancers10080268

**Published:** 2018-08-10

**Authors:** Luciano Pirola, Oskar Ciesielski, Aneta Balcerczyk

**Affiliations:** 1 INSERM U1060, 165 Ch. du Grand Revoyet-BP12, 69921 Oullins, France; luciano.pirola@univ-lyon1.fr; 2 Department of Molecular Biophysics, Faculty of Biology and Environmental Protection, University of Lodz, 90-236 Lodz, Poland; oskar.ciesielski07@wp.pl

**Keywords:** DNA methylation, histone methylation, tumor angiogenesis, metastasis

## Abstract

Approximately 50 years ago, Judah Folkman raised the concept of inhibiting tumor angiogenesis for treating solid tumors. The development of anti-angiogenic drugs would decrease or even arrest tumor growth by restricting the delivery of oxygen and nutrient supplies, while at the same time display minimal toxic side effects to healthy tissues. Bevacizumab (Avastin)—a humanized monoclonal anti VEGF-A antibody—is now used as anti-angiogenic drug in several forms of cancers, yet with variable results. Recent years brought significant progresses in our understanding of the role of chromatin remodeling and epigenetic mechanisms in the regulation of angiogenesis and tumorigenesis. Many inhibitors of DNA methylation as well as of histone methylation, have been successfully tested in preclinical studies and some are currently undergoing evaluation in phase I, II or III clinical trials, either as cytostatic molecules—reducing the proliferation of cancerous cells—or as tumor angiogenesis inhibitors. In this review, we will focus on the methylation status of the vascular epigenome, based on the genomic DNA methylation patterns with DNA methylation being mainly transcriptionally repressive, and lysine/arginine histone post-translational modifications which either promote or repress the chromatin transcriptional state. Finally, we discuss the potential use of “epidrugs” in efficient control of tumor growth and tumor angiogenesis.

## 1. Introduction

In tumors, the synergistic growth of cancerous cells and non-cancerous surrounding vascular structures is necessary to sustain tumor growth. Tumor-derived growth factors stimulate endothelial cells proliferation and neovascularizaton, providing the nutritional supply needed for tumor progression.

Angiogenesis, the process leading to formation of new blood vessels, is an essential physiological and developmental process. Angiogenesis is, however, also a main contributor to tumor growth and the metastatic process [1]. Indeed, tackling tumor angiogenesis has been a major research area to develop strategies aiming at inhibiting of tumor growth by cutting the “supply lines” allowing proliferation of tumor cells [2]. Inhibition of tumor angiogenesis based on administration of recombinant humanized antibodies targeting A-VEGF was the first clinically approved angiogenesis-based therapy and has been used in the treatment of several cancers, including breast, colorectal and lung cancers, but the overall effectiveness of this approach is debated [3]. Other angiogenesis inhibitors, including angiostatin and endostatin, have also been tested in clinical trials [4], without as yet reaching wide marketing approval.

The search for potentially more effective strategies to target cancer growth by inhibiting angiogenesis has revealed that targeting the tumor’s blood vessels epigenetic machinery can be a promising approach to arrest or slow tumor growth [5].

Both DNA methyltransferases (DNMTs), and enzymatic complexes governing histone post-translational modifications are essential epigenetic regulators of gene expression and cell proliferation in the vasculature and in endothelial cells [6]. Studies using histone deacetylases (HDACs) inhibitors demonstrated the role of HDACs in the regulation of tumor cells proliferation [7], endothelial cells proliferation and tumor angiogenesis [8,9].

Recently, substantial research efforts have been directed to investigate the role of DNMTs and histone methyltransferases/histone demethylases in the regulation of tumor angiogenesis and both epigenetic enzymatic systems are potential candidates for epigenetic-based antitumor therapies.

Here, we provide an overview of the current literature and recently published clinical trials supporting the targeting of vascular DNMTs and histone methyltransferases/demethylases as a novel approach to target tumor growth.

## 2. DNA Methylation Profiles in Tumors, Metastasis and Angiogenic Genes

Methylation of DNA, catalysed by the DNA methyltransferases DNMT1, DNMT3a and DNMT3b, plays an important role in regulation of gene expression profile. DNA methylation is a modification of cytosines located within CpG dinucleotides, which receive a methyl group on the 5′ position of the pyrimidine ring. CpG rich regions, called CpG islands are mainly located in gene promoter regions and other genomic regulatory loci, and are usually unmethylated [10,11]. Aberrant methylation occurs in many pathological disorders, including cancer. Methylation events linked to promotion of carcinogenesis and tumor development can consist of (i) DNA hypermethylation of CpGs within gene promoter regions, leading to inactivation of tumor suppressor genes, as well as (ii) DNA hypomethylation, that may activate oncogene transcription (Table 1). The discovery that DNA hypermethylation inactivates tumor suppressors paved the way to the use of DNMT inhibitors in the clinic, as discussed in Section 5.1. DNA hypermethylation constitutes an important mechanism of gene silencing, as almost 60% of human promoters have CpG islands. Additional regulatory mechanisms of gene expression linked to changes in DNA methylation involve methyl-binding proteins (MBDs: MeCP2 (methyl-CpG-binding protein 2), MBD1–6 (methyl-CpG-binding domain proteins 1–6), zinc-finger proteins of Kaiso family) that bind methylated CpGs and exhibit repressive potential, also due to the binding of transcriptional co-repressor molecules [12].

**Figure 1:**
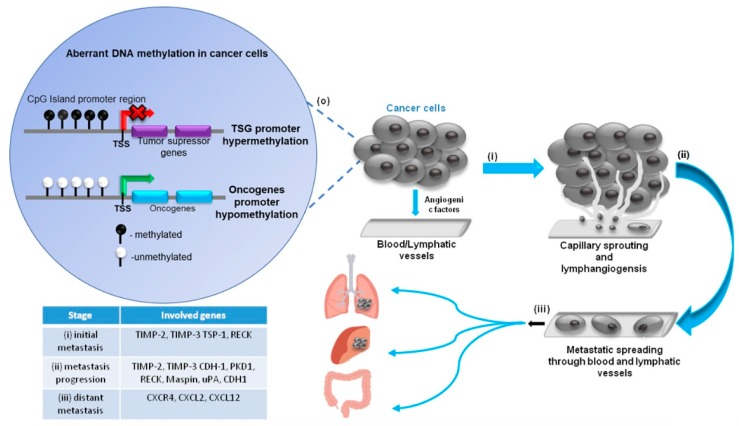
Potential gene targets for anticancer therapy in DNA methylation-guided cancer progression and metastasis. (**o**) Tumor formation as a consequence of aberrant DNA methylation in oncogenes and tumor-suppressor genes and further steps of cancer progression: (**i**) initial metastasis-tumor growth and stimulation of capillary formation, invasion; (**ii**) metastasis progression-intravasation, invasion of cancer cells through the basal membrane into a blood or lymphatic vessel; (**iii**) distant metastasis-extravasation, spreading of cancer cells to nearby lymph nodes, tissues, or organs and formation of distant tumors.

Multiple studies show that changes in DNA methylation are crucial to support the metastatic potential of cancer cells, with metastasis being the main reason of cancer-associated mortality. Angiogenesis, the recruitment of new blood vessels, is an essential component of the metastatic process, as the vessels provide the principal route by which tumor cells exit the primary tumor site and enter the circulation. For many tumors, the vascular density can provide a prognostic indicator of metastatic potential, with highly vascularized primary tumors promoting a higher incidence of metastasis than poorly vascular tumors [23]. Based on cDNA microarrays analysis and genome-wide sequencing techniques, several gene expression signatures were identified. These genes, involved in different steps of metastatic pathway, include (i) metastasis initiation genes—responsible for the regulation of the epithelial–mesenchymal transition (EMT), migration, invasion through tissue barriers, capillary formation and intravasation. Such genes include MMPs, TIMPs (TIMP-2, TIMP-3), TSP-1 and RECK; (ii) metastasis progression genes—ensuring protection in the circulatory system and during extravasation—including TIMPs (TIMP-2, TIMP-3), PKD1, RECK, maspin, uPA, CDH1; (iii) distant metastasis genes, allowing malignant cancer cells to colonize distant organs, which include cytokines, adhesion molecules and proteases, i.e., CXCR4, CXCL2, CXCL12 [24,25] (Figure 1).

Multiple studies indicated that changes in the expression of genes involved in cancer progression are closely related with abnormal DNA methylation patterns. For example, the tissue inhibitor of metalloproteinases (TIMP-2) is suppressed in several types of cancers due to hypermethylation of CpG island in the promoter region, thus increasing the invasive capacity of some cancers (prostate cancer, lymphoid malignancies) [26,27]. TIMP-3 was also found to be suppressed according to the same mechanism, enhancing progression of multiple solid tumors, including breast [28], brain [29], melanoma [30], and gastric cancer [31]. Gene expression alterations linked to aberrant DNA methylation have also been observed for thrombospondin 1 (TSP-1), an endogenous angiogenesis inhibitor [32,33,34,35]. Inactivation of TSP-1, correlating with hypermethylation of its gene promoter, was found in glioblastomas and pancreatic carcinomas, and TSP-1 inactivation in these tumors significantly enhanced their malignancy and metastatic potential, mainly by supporting the vascular invasion process. In all these cases, treatment with demethylating agents (5-aza-cytidine or 5-aza-2′-deoxycytidine) led to demethylation of CpGs, re-expression of the above-mentioned genes, and suppression of cellular invasion [32,33,34]. Also, other genes identified as crucial for cancer progression, including E-cadherin (CDH1), von-Hippel-Lindau (VHL), protein kinase D1 (PKD1), maspin, cysteine-rich protein with Kazal motif (RECK) or urokinase-type plasminogen activator (uPA) were found to be regulated via DNA methylation [35,36,37].

Key genes critical for angiogenesis promotion, (i) vascular endothelial growth factor (VEGF), (ii) its receptors VEGFR1/Flt1, VEGFR2/KDR and VEGFR3/Flt4 and (iii) endothelial nitric oxide synthase (eNOS) also appear to be regulated by their gene promoter methylation status [38,39]. These genes are important regulators not only of endothelial cells, but also support the growth of various solid tumors and leukemias, contributing to the growth of the malignant cells. Both VEGF and eNOS expression were dowregulated via MBD2 binding [39,40]. Using MBD2-null mice, it was reported that abrogation of MBD2 restores eNOS expression and promotes angiogenesis. Given the fact that MBD2 itself does not modify the DNA methylation patterns and appears to be dispensable for normal physiology, these results suggest that MBD2 could be an important epigenetic target to modulate endothelial functions in disease states [39].

Numerous studies analysing DNA methylation patterns, and associated gene expression in tumors, have provided new biomarkers that could be useful in screening for different cancers. Several genes can be used as prognostic indicators. uPA for breast cancer, in which low level of methylation and high expression correlates with a more aggressive histological features in tumor biopsies [41]. NKX2-5, CLSTN1, SPOCK2, SLC16A12, DPYS and NSE1 are candidate biomarkers for prostate cancer, based on their methylation in primary tumors compared to normal adjacent tissues [41]; MAL and TMEM220 for human gastric cancer [42]; SEPT9_v2 promoter for the detection of circulating tumor DNA in breast cancer patients [43]; BNC1 and ADAMTS1 promoter DNA methylation for detection early-stage pancreatic cancers [44].

## 3. Histone Methylation Status as a Key Player in the Regulation of Angiogenesis Process

**Figure 2:**
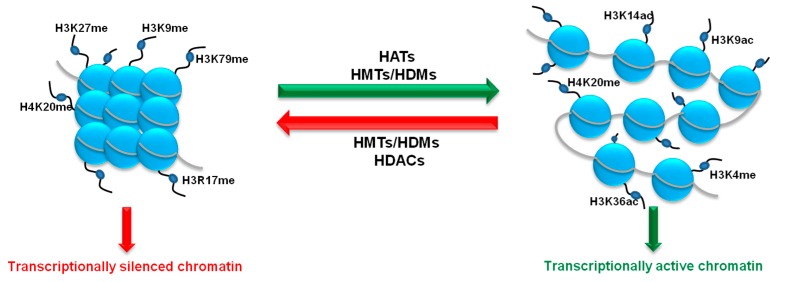
Histone methylation, in association to acetylation, regulate the chromatin transcriptional state. Histone lysine/arginine methyltransferases (HMTs), including EZH2, DOTL1 and G9a promote the formation of condensed and transcriptionally repressed chromatin. Histone demethylases (HDMs) in concert with histone acetyltransferases (HATs) and the histone H3K4 methylase SET7 promote the transcriptionally active chromatic state. Histone deacetylases (HDACs) contribute to transcriptional silencing (not discussed in this review).

Histone acetylation and methylation are the most widely studied histone PTMs (post-translational modifications), the interplay of which dictates accessibility of chromatic regions and, consequently, gene expression. While histone acetylation-taking place on the four core histones and at multiple lysine residues-mainly conveys a transcriptionally permissive signal [45], methylation is either transcriptionally permissive or repressive depending on the lysine or arginine residues being targeted. Transcriptionally permissive methylations include H3K4me, H3K36me and H4K20me, and repressive modifications take place on H4K20me, H3K9me, H3K27me and H3K79me [46] (Figure 2). Similarly, arginine methylation can either promote or repress the building of transcriptional complexes [47].

Addition/removal of the methyl group to/from the histone tails does not affect overall charge of the proteins but changes their hydrophobicity and influences their affinity to selected proteins including transcription factors, thus efficiently contributing to modifications of multiple metabolic processes, including angiogenesis and carcinogenesis [48].

### 3.1. Histone Methylating Enzymes (HMTs)

#### 3.1.1. Targeting the Polycomb Repressive Complex 2 in Cancer

Histone H3 lysine 27 trimethylation, mediated by the histone methyltransferase Enhancer of Zeste Homolog 2 (EZH2), which is the catalytic component of the polycomb repressive complex 2 (PCR2), is a central negative regulator of gene expression. Retention of H3K27 trimethylation during mitosis also participates to the persistent transmission of a transcriptionally repressive state of its target chromatic regions throughout cell division [49].

EZH2 has been shown to participate in the regulation of angiogenesis. In human umbilical vein endothelial cells (HUVECs), gene silencing of EZH2 impaired cell adhesion, migration, and in vitro capillary tube formation, suggesting that H3K27 trimethylation contributes to the regulation of angiogenesis [50]. In tumor cells, EZH2 mediates the silencing of anti-metastatic genes, including E-cadherin and tissue inhibitors of metalloproteinases, favoring cell spreading and anchorage-independent growth [51]. Additionally, EZH2 also promotes tumor angiogenesis and EZH2 inhibition has been shown to inhibit the differentiation of cancer stem cells into endothelial cells [52].

The increased expression of EZH2 has been shown in several cancers, including metastatic prostate [53] and breast cancer [54], and is a predictor of a poor clinical outcome [54,55]. Interestingly, tumor progression and poor clinical outcome have also been observed upon EZH2 overexpression in the tumor vasculature rather than in the cancerous cells [5].

Endothelial overexpression of EZH2 has been shown to be dependent on a paracrine VEGF stimulation, promoting angiogenesis via histone H3K27 trimethylation on the vasohibin1 (vash1) promoter. Transcriptional silencing of EZH2 in the tumor-associated endothelial cells allowed re-expression of vasohibin1 and inhibition of tumor angiogenesis. In two independent murine models of orthotopic ovarian carcinoma, established by injection of HeyA8 or SKOV3ip1 cells human ovarian carcinoma cell lines, siRNA-mediated inhibition of human EZH2 led to only a mild tumor inhibition. Conversely, siRNA-mediated targeting of murine EZH2, acting on the tumor vasculature but not the tumor cells, had a stronger inhibitory effect on tumor growth, and the simultaneous addition of murine and human siRNA lead to a maximal inhibitory affect, supporting the notion that EZH2 targeting can be a therapeutic approach acting on both the tumor cells and the vasculature [5].

In gliomas, a predominantly lethal class of tumors, a major feature is the occurrence of angiogenic regions in which glioma stem cells (GSCs) develop a proneural profile and hypoxic regions associated with mesenchymal GSCs. Tumor cells associated to the vascularized region of glyomas harbor an activated EZH2, while GSCs developing into the hypoxic part of the tumor express BMI1 (a component of the Polycomb Repressor Complex1), dual pharmacologic inhibition of EZH2 and BMI1 in cell culture and a preclinical model was more effective than the use of a single pharmacological agent [56].

The link between EZH2 overexpression and tumor progression and aggressiveness might suggest that, conversely, overexpression of histone demethylases might confer tumor suppressive properties to tumor cells or be able to suppress angiogenesis. The methyltransferase activity of PRC2 is balanced by three major demethylases: JHDM1D, KDM6a, and KDM6b.

The histone demethylase JHDM1D, a member of the Jumonji family of histone demethylases (also known as KDM7A) specifically demethylates the repressive marks of mono- and di-methylated histone H3K9 and histone H3K27, counteracting epigenetic gene regulation of HMTs [57]. In cell culture of mouse and human tumor cells, the expression of JHDM1D was elicited by long-term nutrient starvation. Overexpressed JHDM1D in tumor cells undergoing nutrient starvation was shown to have a tumor-suppressive role via regulation of angiogenesis. Xenografts of tumor cells overexpressing JHDM1D lead to a decrease of proangiogenic factors, including VEGF-B and angiopoietin, resulting in inhibition of tumor growth [58].

Taken together, studies demonstrating that histone methyltransferase pharmacological inhibition and, conversely, KDM7A overexpression lead to inhibition of tumor growth by acting on tumor-associated angiogenesis indicate that therapeutic targeting of components of the epigenetic machinery may be a promising strategy to block tumor growth.

#### 3.1.2. G9a Histone Methyltransferase

Euchromatic lysine methyltransferase 2, also known as G9a is a lysine methyltransferases (KMT) that methylates the 9 and 27 lysine residues of histone H3, resulting in transcriptional repression. The role of G9a in cancer is well established, as it was found to be post-transcriptionally upregulated in response to hypoxia [59] and to act as a promoter of tumorgenesis by silencing tumor suppressor genes [60,61]. The action of G9a is also essential to maintain a malignant phenotype. Recent studies suggest that G9a is required to sustain cancer cell proliferation and survival [62,63]. The knockdown of G9a in hepatocellular carcinoma cells resulted in decreased cell growth and sphere formation [64]. Besides being required for cancer cell proliferation, there is growing evidence proving that G9a may also be essential to drive tumor angiogenesis.

G9a was found to be overexpressed in cervical cancer cells, compared to normal cervix and cancer precursors. To evaluate the effect of G9a on the expression of angiogenic factors expression, SiHa cells were treated with BIX-01294, a G9a HMT inhibitor, for 24 h. In BIX-01294 treated cells, several pro-angiogenic factors, including: VEGF, interleukin-8 and angiogenin were significantly inhibited. The role of G9a in angiogenesis was further investigated in vitro*.* Conditioned media from BIX-01294 treated cells reduced endothelial cell proliferation, migration and permeability. Moreover, such treatment also impaired the number of polygonal vascular tube formations of endothelial cells. Taken together, these results suggest that G9a HMT is an active player of angiogenesis regulation in cervical cancer [65]. The inhibition of G9a via BIX-01294 was also shown to impair the stability of hypoxia-inducible factor-1α (HIF-1α, one of the key regulators of tumor growth and angiogenesis) by decreasing gene expression of proline hydroxylase 2 (PHD2) and the half-life of HIF-1α in HepG2 human hepatocellular carcinoma cells [66].

Endothelial cell proliferation is essential for angiogenesis, thus the role of G9a in the regulation of endothelial cells proliferation was recently assessed using both pharmacological and transcriptional inhibition of the enzyme. Pharmacological inhibition of G9a HMT activity by BIX-01294 treatment, as well as shRNA-mediated transcriptional inhibition in HMEC-1 cells resulted in decreased cell proliferation and induction of cell cycle arrest in G1 phase. This proves that inhibitors of G9a not only act as anti-carcinogenic agents in cancer cells, but can also be used in the treatment or prevention of tumor neovascularization [67].

Higher G9a expression is often correlated with decreased patient survival. The correlation between G9a expression and survival rate was recently evaluated in a group of cervical cancer patients. Analysis showed that higher G9a expression correlated with poorer survival time [65]. As G9a not only increases tumor cell proliferation, but also promotes angiogenesis, this HMT emerges as important target in cancer therapies.

#### 3.1.3. DOT1L and Other Histone Methyltransferases

The enzymatic action of DOT1L, a H3K79 histone methyltransferase is also required for angiogenesis. Silencing of DOT1L in HUVECs resulted in decreased cell viability, migration, tube formation and capillary sprout formation. It also reduced the formation of functional vascular networks in a matrigel assay. DOT1L acts in concert with ETS-1 transcription factor to promote the expression of VEGFR2, which in turn activates the ERK1/2 and AKT signaling pathways, thus promoting angiogenesis. The pro-angiogenic role of DOT1L, was also supported from in vivo spheroid assay in mice, where knockdown of DOT1L resulted in reduction of the vascular density [68]. A further KMT that was recently shown to be involved in angiogenesis is SET7, that acts in concert with GATA1, a key regulator of angiogenesis. The SET7-GATA1 interaction results in VEGF transcriptional up-regulation and promotion of angiogenesis. Knockdown of SET7 in breast cancer cells inhibited their growth, both in vitro and in vivo, and also reduced HUVECs proliferation, migration, tube formation and therefore tumor angiogenesis by decreased VEGF secretion in breast cancer cells [69].

## 4. Histone Demethylases

Overexpression of the H3K9/H3K27 demethylase JHDM1D, leading to demethylation of epigenetically repressive marks, has a comparable action to targeting the HMT EZH2, as both interventions lead to H3K9/H3K27 demethylation and transcriptional activation of anti-angiogenic pathways. A second KDM, JARID1B, a member of the JmjC/ARID family of histone demethylases with substrate specificity towards tri- and di-methylated histone H3 lysine 4 (H3K4Me2/Me3), a methylation taking place on transcriptionally active chromatin, has also been shown to possess anti-angiogenic properties. JARID1B and LSD1 (lysine specific demethylase 1, demethylating H3K4me1) have been shown to sequentially demethylate H3K4. One of the cellular signaling pathways targeted by, and inhibited by, the JARID1B/LSD1 sequential demethylations is the CCL14 chemokine pathway that controls cell migration and angiogenesis. Therefore, repression of CCL14 suppressed the angiogenic and metastatic features of breast cancer cells in vivo [70].

The above study demonstrates that promoting the de-methylation of repressive marks has a comparable effect as inhibiting the HMT EZH2. Similarly, inhibition of H3K4 methylation, an activatory mark, through targeting of their JARID1B/LSD1/NuRD complex also display anti- tumor efficacy by blocking the CCL14 chemokine pathway controlling cell migration and angiogenesis [70]. Therapeutic targeting of components of the histone demethylases complexes may therefore be a promising strategy to block tumor growth.

## 5. Methylation-Focused Epidrugs in Tumor Angiogenesis Control: Drug Candidates and Ongoing Clinical Trials

The reversibility and dynamic nature of epigenetic events in tumor growth, in contrast to the genetic tumor mutations, makes them more suitable for therapeutic strategies. The (pre)clinical studies on epigenetic-based therapy designed for cancer treatment are mainly focused on DNMT inhibitors (DNMTis) and, relatively to histone posttranslational modifications, on HDAC inhibitors (HDACis). Nevertheless, the growing evidence supporting a key role of histone methylation processes in tumorgenesis as well as tumor angiogenesis promoted the more recent development of therapeutic molecules targeting histone methyltransferases and demethylases [71].

Early clinical trials using DNMTis and HDACis can be traced back to more than half a century ago [72], whereas pre-clinical and clinical trial testing of epigenetic drugs targeting more recently recognized epigenetic mechanisms: (i) bromodomain and extraterminal protein (BET) inhibitors, (ii) lysine-specific demethylase 1A (LSD1/KDM1A) inhibitors, (iii) histone methyltransferase (HMT) and (iv) protein arginine methyltransferase (PRMT) inhibitors, was only started within last ten years. The total number of trials evaluating DNMTis and HDACis can be counted in the hundreds. The total number of trials evaluating epigenetic drugs acting via the above-mentioned newer mechanisms constituted just a small percentage of these totals. Only 45 trials with BET inhibitors, 12 with LSD1A/KDM1A inhibitors (currently in phase I/II) and 12 with HMT/PRMT inhibitors have been conducted so far [73,74]. 

### 5.1. DNA Methyltransferase Inhibitors (DNMTis)

#### 5.1.1. Azacitidine and Decitabine—The Fundamental Hypomethylating Agents

The first epigenetic drugs that have been approved for cancer chemotherapy, azacytidine and decitabine, are demethylating agents (Table 2). These nucleoside cytidine analogues, once entered into the cell, are phosphorylated by cellular kinases and then incorporated into DNA. Azacytidine (5-azacytidine, AZA; Vidaza^®^), when incorporated into DNA, acts as an irreversibe inhibitor of DNMT1. Because azacytidine is also incorporated into RNA, it acts on multiple molecular levels of cellular metabolism by: (i) inhibiting DNA synthesis, (ii) inducing dysfunction of RNA (ribosomal disassembly and defective tRNA functioning), resulting in the inhibition of protein synthesis [75]. Unlike 5-azacytidine, decitabine (5-aza-2′-deoxycytidine, DAC; Dacogen^®^) is incorporated only into DNA. Due to its irreversible mechanism of inhibition of DNMT1, decitabine leads to the rapid inactivation of the methyltransferase, making it unavailable for further methylation, thus resulting in generalized hypomethylation of genomic DNA, also on replicating nascent molecules [76,77]. Both 5-azacytidine and decitabine can reactivate epigenetically silenced tumor suppressor genes, including cell-cycle inhibitors (p14ARF, p15INK4b, p16INK4a, p21Cip/WAF, p27Kip1), pro-apoptotic genes (ARHI, APC, RASSF1A, HIC1), DNA repair genes (BRCA1, GSTP1, hMLH1, MGMT), genes related to metastasis (CDH1, DAPK, maspin, TIMP-3, TSP1, VHL) or differentiation markers (e.g., RARβ2) that are silenced by methylation of CpG islands on their promoters, thus decreasing tumor growth [78]. Both 5-azacytidine and decitabine are inactivated by cytidine deaminase, which catalyzes their deamination to uridine analogues [79].

Despite promising in vitro data showing the antiproliferative potential of cytidine analogues (exhibiting the greatest cytotoxicity during the S-phase of cell cycle by arresting DNA replication) towards different tumor cell lines as well as an angiostatic potential, through suppression of key genes promoting angiogenesis, e.g., TSP-1, ICAM-1 or RECK, clinical trials revealed poor efficacy of decitabine as well as azacytidine in solid tumors, mainly due to their (i) high clearance rate in vivo (the terminal elimination half-life (*t*_1/2_) is 37–47 min for decitabine and 1.5–2.3 h for azacitidine), and (ii) instability of the compounds in the acidic conditions of the tumor environment [77,80]. The drugs gained clinical approval mainly for hematological applications such as myelodysplastic syndromes and leukemias (Table 1). In case of solid tumors, such as colon, bladder, breast cancer, lung cancers, the most promising effects have been obtained in combined clinical therapies with histone deacetylase inhibitors/cisplatin: azacitidine+valproic acid (NCT00496444), azacitidine+entinostat (NCT00101179), decitabine+vorinostat (NCT00357708, NCT00479232), decitabine+valproic acid (NCT00075010, NCT00109824), decitabine+romidepsin (NCT00037817, NCT00114257). (Trial codes have been retrieved from the ClinicalTrials website hosted by the U.S. National Library of Medicine [73].

The pre-clinical research and ongoing clinical trials aim to search and test for different epigenetic drugs to increase the efficiency of treatment by testing (i) novel chemotherapeutic cocktails and (ii) new cytotoxic drugs. The family of DNA hypomethylating agents targeting tumor angiogenesis is rapidly expanding. The list of DNMTis that promisingly passed in vitro and in vivo preclinical tests, but have not completed yet the regulatory procedures for clinical testing, include (i) novel nucleoside analogs; (ii) antisense oligonucleotides; (iii) low molecular weight molecules and also some (iv) natural compounds (Table 3). 

#### 5.1.2. Zebularine: A Novel DNA Methyltransferase Inhibitor

Zebularine (Zeb) is a novel nucleoside analog acting as *DNMTi* that is currently undergoing clinical testing. As compared to azacitidine and decitabine, Zeb is characterized by a reduced toxicity towards normal cells and increased pharmacokinetic stability that allows switching from the intravenous administration of the drug in favor of the oral application, yet without losing its demethylating capabilities [99,100]. Like azacitidine, Zebularine is a substrate of uridine-cytidine kinase, and is thus incorporated into DNA [101]. The molecular mechanism for ZEB inhibitory action is based on the formation of a reversible covalent bond with DNMT1. Nevertheless, the inhibitory activity of Zeb is not limited to DNMT1 only. Zeb is also a strong inhibitor of cytidine deaminase [102,103]. Research performed on tumor-conditioned HUVECs, revealed the angiostatic potential of zebularine via induction of ICAM1 expression and restoration of leukocyte adhesion to endothelial cells [85], as well as restoration of the expression of the anti-angiogenic genes TSP1, JUNB, and IGFBP5 [33]. In vivo studies validated ability of Zeb for inhibition of tumor vascularization leading to reduction of colon cancer and melanoma growth [33]. Antiangiogenic potential was also observed in testing the decitabine antimetabolite guadecitabine, that was shown to reactivate a few epigenetically silenced tumor suppressor genes: CDKN2A, DLEC1, RUNX3 and reduce tumor growth via inhibition of angiogenesis in a hepatocellular carcinoma model [83,84].

#### 5.1.3. Antisense Oligonucleotides Inhibitors of DNMTs

Short antisense oligodeoxynucleotide sequences complementary to the 3′ untranslated region of the DNMT1 methyltransferase mRNA suppresses DNMT1 gene expression, leading to a DNMT1 knockdown. Promising results were obtained in clinical testing of the second-generation antisense oligonucleotide MG98 (NCT00003890), especially towards solid tumors treatment (renal carcinoma), [86,87].

#### 5.1.4. Low Molecular Weight Inhibitors of DNMTs

The family of small molecules involved in modification of the DNA methylation pattern is very heterogeneous at the structural level, mode of action and substrate specificity. In several instances, DNA demethylation is not the only exerted biological effect. Hydralazine and disulfiram block the enzymatic activity of DNMTs in a competitive manner by decreasing the affinity of DNMTs for *S*-adenosylmethionine (SAM) and nucleic acid, directly binding to CpG-rich sequences, whereas RG108 (a phthaloyl tryptophan derivative) directly inhibits DNMT1 through binding to the active site of the enzyme. These molecules present anticancer and antiangiogenic abilities by inducing re-expression of various tumor suppressor genes, e.g., RAR-β, p16 (hydralazine, RG108, disulfiram), affecting VEGF synthesis and release (hydralazine) or metalloproteinase activity (disulfiram) [88,91,92,104]. Additionally, it was found that RG108 does not affect methylation at centromeric satellite repeats contrary to disulfiram and hyralazine, that affect centromere methylation leading to centromere instability [104,105]. Due to their promising preclinical data, the drugs are extensively tested in multiple clinical trials (e.g., NCT00404326, NCT00395655, NCT00404508).

#### 5.1.5. Natural Compounds with DNMTs Inhibitory Activity

The growing interest in the pharmaceutical values of natural products has, in several instances, revealed their ability to modulate the epigenome, also acting on the DNA methylation level, e.g., curcumin, (−)-epigallocatechin-3-gallate (EGCG), psammaplin A. These so-called “epi-nutrients”, have become valuable molecules for cancer chemoprevention strategies and supporting elements of regeneration of the organism after chemotherapy. The above-mentioned compounds have been shown to inhibit DNMT1 (curcumin with an IC_50_ of 30 nM [106]; EGCG with a K_i_ of 6.89 μM [107]) and induce re-expression of hypermethylated tumor suppressor genes, and are currently employed in clinical trials on pancreatic cancer (NCT00094445) and non-metastatic bladder cancer (NCT00666562).

Curcumin displays multiple antiangiogenic properties, including dowregulation of the transcription factors NF-κB and STAT3; proangiogenic factors VEGF, bFGF, COX-2; inhibition of endothelial cell migration and invasion [93,108,109] as well as antiproliferative and pro-apototic effects on tumor cells [110,111]. A potential drawback is that curcumin is poorly absorbed from intestine, and its systemic bioavailability after oral feeding is relatively low. Nevertheless, as an ingredient of turmeric, curcumin has exhibited beneficial health effects, related to its anti-inflammatory, hypoglycemic, antioxidant, wound-healing, and antimicrobial activities [112].

Psammaplin A (PsA) is a marine natural compound extracted from the *Psammaplinaplysilla* sponge. Chemically, PsA is a bisulfide bromotyrosine derivative, targeting both DNMT1 as well as HDACs [113]. In vitro studies have shown that PsA exerts strong cytotoxic effects in the human tumor cell lines A549, MCF7, and W138 and reduces tumor cell growth in a A549 lung xenograft mouse model [114]. Recent findings reveal that PsA is sufficient to overcome multidrug-resistant cancer via SIRT1-mediated autophagy in doxorubicin-resistant MCF-7/adr breast cancer cells, indicating that PsA has therapeutic potential for clinical use [115].

Several catechol-containing polyphenols, such as tea catechins (catechin, epicatechin) and bioflavonoids (quercetin, genistein) were also identified as DNMT inhibitors. The strongest inhibitory activity against carcinogenesis and angiogenesis can be attributed to the major green tea polyphenol, (−)-epigallocatechin-3-gallate (EGCG), as confirmed in different experimental models including non-small cell lung carcinoma cells (NSCLC), A549 lung carcinoma xenografts, E0771 mouse breast adenocarcinoma and oral squamous carcinoma cells [116,117,118,119]. EGCG exhibits strong hypomethylating potential and also inhibits tumor angiogenesis in xenograft models through down-regulation of vascular endothelial growth factor A (VEGFA) and hypoxia inducible factor 1 alpha (HIF1α) in endometrial cancer [116].

### 5.2. Hypomethylating Agents (DNMTis) in Co-treatment with Methyl Group Donor (SAM) in the Prevention of Tumor Progression via Tumor Angiogenesis Inhibition

Although a lot of studies recommend using hypomethylation agents for cancer treatment, some research also suggests the inclusion of hypermethylating agents in therapies against cancer and metastasis. *S*-adenosylmethionine (AdoMet, also known as SAM) is the main biological methyl donor synthesized from methionine and ATP in the reaction catalysed by methionine adenosyltransferase (MAT) in all mammalian cells, and most abundantly in the liver. Numerous studies confirm that the efficiency of DNA methylation is directly dependent on the intracellular concentration of SAM. It was found that exogenous treatment with SAM caused hypermethylation of DNA and inhibited DNA demethylation either by enhancing DNA methyltransferase activity or by inhibiting its demethylation [120,121].

Detailed analysis of the effects of SAM on cellular metabolism revealed that the compound promotes apoptosis in tumor cells originating from gastric, colon, liver or prostate cancer, whereas is significantly less harmful for normal cells [120,122,123]. In line with these findings it was shown that, at the molecular level, SAM induces uPA gene silencing via hypermethylation of its gene promoter that results in the inhibition of tumor cell invasion in vitro, and metastasis and cancer growth in in vivo conditions [37,120]. It was also shown that SAM effectively induces DNA methylation on oncogenes involved in cancerogenesis, such as S-myc and H-Ras, leading to their inactivation and also stimulates silencing of expression of critical tumor growth-/tumor angiogenesis- promoting genes (MMP2, MMP9, VEGF, PAI-1, TGF-β, RUNX2) [122,124]. Collectively, these data provide support that SAM can serve as a potential therapeutic reagent for anticancer therapy provided that the tumorigenicity is linked to overexpression of oncogenes, as SAM administration would be detrimental in tumors caused by loss of tumor suppressors.

**Figure 3:**
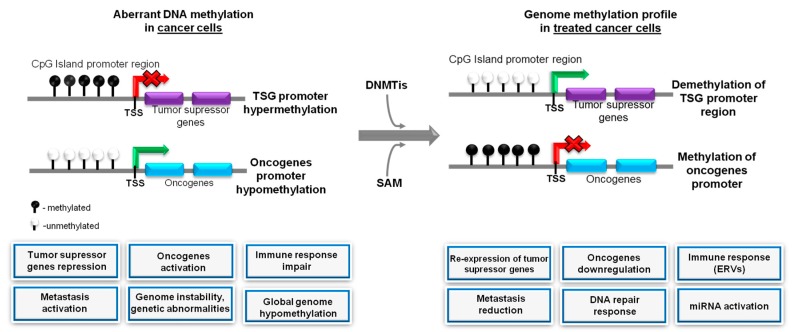
Schematic anti-cancer mechanism of the targeted therapy based on DNA inhibitors and SAM treatment/positive and negative effects of epidrug action*.* In the cancer genome, DNA hypermethylation and hypomethylation causes the inactivation of tumor suppressor genes and activation of oncogenes, respectively. DNMT inhibitors block hypermethylation of DNA, hence decreasing methylation the promoters of tumor suppressor genes causing upregulation of their expression. On the other hand, SAM can block the activation of oncogenes and proto-oncogenes. Taken together, the combination of these two agents is likely to combat the DNA abnormalities of gene expression seen in cancer.

The profiles of DNA methylation in cancer cells are significantly changed in comparison to normal cells, Table 1. Apart from the global hypomethylation of genome, aberrations in methylation of oncogenes/proto-oncogenes as well as tumor suppressor genes, crucial for tumor growth and progression are observed. Promising results of anticancer therapies using DNA methylation inhibitors, revealed, at the same time the drawbacks of such treatments. For example, hypomethylating agents treatment results in a broad landscape of demethylated gene promoters on tumor suppressor genes but also on important genes involved in migration and invasion responsible for cancer metastasis, the most morbid aspect of cancer [125]. Encouraging data coming from SAM supplementation studies prompted into applying a combined treatment using both hypo- and hyper- methylation agents, Figure 3.

Studies testing whether SAM antagonizes the prometastatic effect exerted on tumor cells by hypomethylating agents (Decitabine, Azacitidine) gave positive results. In the combined treatment, it was found that SAM reverses the prometastatic effects of Azacitidine and also augments its tumor antigrowth action [126,127]. These data implicate that SAM mechanism of action in a co-treatment involves partial blocking of the DNA demethylation induced by Azacitidine, yet excluding tumor suppressor genes. However, further studies may be needed before a clinical attempt of usage both inhibitors/activators of DNA methylation, as it was found that combined treatment may also cause an increment in genome fragility [127].

### 5.3. Epidrugs Modulating Angiogenesis Process via the Histone Methylation Status

The search of therapeutic compounds that selectively inhibit histone methyltransferases (HMTs) and demethylases (DMTs) is still at the beginning and offers a big space for discovery and pharmacological interventions. Methylation level of arginine and lysine residues plays an important role in the regulation of metabolism, cancerous growth and endothelial cell function [67,128,129,130,131] due to its effect on chromatin reorganisation that limits the access of the transcriptional machinery to DNA.

#### 5.3.1. Histone Methyltransferase Inhibitors (HMTIs)

Up to date, 9 arginine human lysine methyltransferases (PRMTs) and more than 50 lysine human methyltransferases (KMTs) have been reported. Histone methyltransferases are involved in the regulation of angiogenesis and tumor growth. The most relevant ones, including DOT1L, EZH2, Set7, SUV3-9H or G9a have been described in Section 3.1. Several molecules targeting HMTs have been tested in vitro and in vivo and a selection of these inhibitors has now proceeded into clinical testing. First-line inhibitors of histone methyltransferases were analogues of SAM (*S*-adenosylmethionine) and SAH (*S*-adenosyl-L-homocysteine), nevertheless these compounds are not solely specific to HMTs as they also affect other enzymes using AdoMet as methyl group donor, e.g., DNA methyltransferases. More specific compounds have thus been developed, as presented below.

##### Inhibitors of histone lysine methyltransferases (HKMTs)

**Figure 4:**
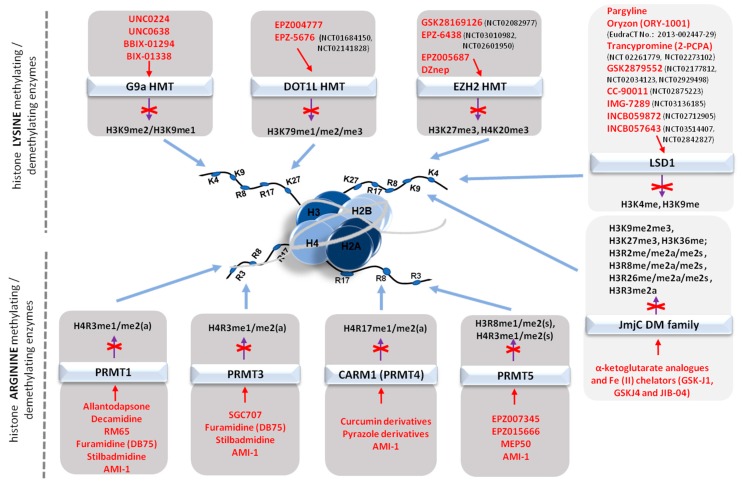
Schematic representation of the modifications of histone core proteins methylation status by lysine and arginine methyltransferases/demethylases inhibitors*.* The methylation status of histones is modified by specific molecules with approved inhibitory abilities verified in multiple in vitro/in vivo studies or tested in clinical trials (the number of trials has been included in brackets) toward lysine and arginine histone methyltransferases/demethylases. Inhibitory, biochemical and biological properties of indicated inhibitors (distinguished by red font) are presented in the main text.

As the angiogenic process can be regulated at multiple levels including proliferation, migration and the ability of endothelial cells to produce capillary-like structures, several molecules affecting histone methyltransferase activity have been introduced with very promising results. A first set of tested epidrugs include compounds specifically inhibiting enzymes abundantly overexpressed in multiple types of cancers as breast, prostate, lung or blood, with nevertheless only a few molecules in a clinical phase of testing (Figure 4).

The EPZs class of compounds, including EPZ004777 and its derivative EPZ-5676, targeting DOT1L—a methyltransferase that regulates angiogenesis via VEGFR2 and is also involved in control of proliferation, differentiation and embryogenesis [68]—have been positively validated in a cellular model for selective killing of MLL-rearranged leukemia cells in culture, while having a significantly less toxic effect on non MLL-rearranged cells [132]. Both EPZs molecules use SAM, and alternatively also SAH, as a cofactor. Better pharmacokinetic parameters for EPZ-5676 (Pinometostat) as compared to EPZ004777 have been reported. In particular, improvements regarding oral bioavailability, qualified the inhibitor for phase I clinical testing (NCT01684150, NCT02141828) for the treatment of leukemias, AML and ALL with translocation of the MLL gene [133,134].

Another important pharmacological target for anticancer therapy is EZH2, a component of the polycomb repressive complex, responsible for the silencing of tumor suppressor genes in cancer cells as well as a promoter of tumor angiogenesis via dowregulation of vasohibin 1 expression (VASH1), a soluble inhibitor of tumor angiogenesis [135,136]. The most studied inhibitor of EZH2 in cellular models is 3-deazaneplanocin A (DZnep), that reduces enzyme expression and inhibits the repressive methylations of H3K27me3 and H4K20me3. The consequences of DZnep treatment are manifested by cell cycle arrest and induction of apoptosis, as it was found that the inhibitor affects cell cycle regulators by increasing p21, p27 and FBXO32 expression [137]. Pre-clinical studies showed that DZnep is able to silence several anti-metastatic genes (e.g., E-cadherin and tissue inhibitors of metalloproteinases such as TIMP-3), thereby favoring cell invasion and anchorage-independent growth. In addition, DZnep was able to inhibit cancer cell invasion and tumor angiogenesis in prostate and brain cancers, respectively. It was found that, at tumor-inhibiting doses, DZnep is not harmful for non-transformed cells [51,79,138]. Extensive studies on the pharmacokinetics of EZH2 inhibitors allowed to identify a selection of molecules, with better bioavailability and higher specificity that minimize off-target effects, directed to the conserved Set-domain of EZH2 that exhibit methyltransferase activity and has been identified as a mutated catalytic domain in human cancers, with more than 50 mutations reported. A detailed study reported that EPZ005687, a SAM-competitive inhibitor, can inhibit H3K27 methylation mediated by the EZH2 mutants Y641 and A677, and has also been shown to selectively kill lymphoma cells that are heterozygous for one of these EZH2 mutations [139]. Two other EZH2 inhibitors: GSK2816126 and EPZ-6438 are currently in phase I and II clinical trials, respectively. GSK2816126 that shows more than a 1000-fold higher selectivity of EZH2 than for other 20 human methyltransferases containing SET or non-SET domains, and effectively inhibits the proliferation of EZH2 mutants in diffuse large B-cell lymphoma cell lines and the growth of EZH2-mutant diffuse large B-cell lymphoma xenografts in mice [140]. Currently the compound is tested for transformed follicular lymphoma, other non-Hodgkin’s lymphomas, solid tumors and multiple myeloma (NCT02082977). EPZ-6438, another EZH2 inhibitor also known as Tazemetostat, selectively kills NHL cells bearing mutations within EZH2, and it has minimal effects on the proliferation of EZH2 wild-type NHL cells. EPZ-6438 is currently undergoing a phase I trial in patients with advanced solid tumors or with relapsed or refractory B-cell lymphoma (NCT03010982) and is also in phase II of tests for adult subjects with INI1-negative tumors or relapsed/refractory synovial sarcoma (NCT02601950) (clinical trial identifiers allow to retrieve the trial registration on https://www.clinicaltrials.gov/ [73]).

In tests performed on cellular models, promising results have been shown by compounds inhibiting G9a activity, an enzyme belonging to the SUV39 family: these compounds include BIX molecules (BIX-01294 and BIX-01338), that are non-competitive inhibitors for the SAM co-substrate and reduce H3K9me2 levels; UNC0224 and UNC0638 specifically target GLP protein, Set7/9 and Set8 [141]. Nevertheless, none of these inhibitors have yet been entered clinical testing.

##### Inhibitors of histone arginine methyltransferases (PRMTs)

Multiple studies on the role of arginine methyltransferases in cellular metabolisms and signalling pathways support their involvement in cancer development, including; (i) cell proliferation—PRMT1, PRMT2, CARM1, PRMT5, PRMT6; PRMT8, PRMT9; (ii) growth stimulation—PRMT1, PRMT2, CARM1, PRMT6; (iii) invasion and metastasis-PRMT1, CARM1, PRMT6; PRMT7, and (iv) angiogenesis-PRMT6 [142]. Overexpression or enhanced activity of PRMTs has been recognized in multiple type of cancers (breast, prostate, lung, colon, leukemias), cardiovascular diseases, but also in neurodegenerative diseases (Huntington’s disease (PRMT5) and Alzheimer disease (PRMT5)) [129,142,143,144,145]. The specific role of PRMTs in cancer pathology, however, is still insufficiently described as well as the development of specific inhibitors. None of the few PRMTs inhibitors known (Figure 4) have as yet been approved for clinical testing, and these molecules have been tested mainly at biochemical level and in cellular models. Targeting PRMTs opens big opportunities for studies in the future years. Additionally, for some PRMTs, including PRMT2, PRMT7, PRMT8 and PRMT9, specific inhibitors have not been reported yet.

The best known PRMTs inhibitor, which also shows a broad substrate spectrum including PRMT1, PRMT3, PR, including angiogenesis and carcinogenesis MT4, PRMT5 and PRMT6 is AMI-1 (IC_50_ of 55 μM in a TR-FRET assay). The compound is a non-SAM-competitive inhibitor and affects lysine methyltransferases activity only to a very low extent. It was also reported that AMI-1 is able to inhibit the methylation level of exogenous nucleolar protein 3 (NOL3) and endogenous Sam68 protein in HeLa cells and suppresses the effects of PRMT1 and CARM1 on nuclear receptor dependent transcriptional activation in MCF7 cells [146]. It was reported that AMI-1 inhibits the growth of solid tumors and reduces cervical cancer cell proliferation, colony formation and promotes cell apoptosis, as well as inhibits the ability of endothelial cells for capillary-like tube formation network in vitro [130,147].

Virtual screening and multilevel biological evaluations allowed to identify several compounds specifically targeting PRMT1, including: allantodapsone (IC_50_ of 1.7 μM in a DELFIA assay) [148], RM65 (IC_50_ around 55 μM) [149], stilbamidine (IC_50_ around 57 μM in a filter-binding assay) [150], furamidine also known as DB75 (IC_50_ around 9.4 μM assessed in filter-binding assay) [149], decamidine (IC_50_ of 13 μM validated in secondary orthogonal assays) [151], showing inhibition of cellular H4R3 methylation in several cell types: HepG2 hepatocellular carcinoma cells, MCF7 breast cancer cells, LNCaP prostate cancer cells, THP1 leukemic monocytes and acute myeloid leukemia cells MOLM-13 [143,152]. The indicated above molecules are highly specific to PRMT1, yet display also a lower affinity towards other methyltransferases, including angiogenesis and carcinogenesis., stilbamidine also inhibits PRMT3, PRMT5 and PRMT6, whereas DB75 presents 42-fold lower affinity to CARM1, more than 30-fold to PRMT5 and 30-fold to PRMT6 than to PRMT1 [143,149,150].

Regarding other arginine methyltransferases, the best characterized cell-active allosteric inhibitor of PRMT3-SGC707—was proven to inhibit the methylation of both endogenous and exogenous H4R3 and bind to overexpressed PRMT3 in the embryonic kidney cell line HEK293 and lung cancer cell line A549, with a K_d_ at 85 nM (using an isothermal titration calorimetry assay) [153,154]. CARM1 activity, also recognized as a transcriptional co-activator, was extensively studied in the presence of curcumin derivatives that were identified as potential inhibitors (IC_50_ of 8.6 μM; using a reporter gene assay), as well as pyrazole inhibitors (IC_50_ of 1.8 μM) [155]. Based on decreased symmetric dimethylation of H3R8 and H4R3 in a set of biochemical assays (TR-FRET, SPA) novel PRMT5 inhibitors were identified e.g., EPZ007345, EPZ015666 or MEP50 [156,157,158]. Presently, the several PRMTs inhibitors identified described above have shown anti-tumor efficacy in cell culture studies, and further validation of their efficacy in preclinical animal models will be necessary prior to the testing of these molecules in clinical trials.

#### 5.3.2. Histone Demethylase Inhibitors (KDMIs)

The epigenetic abnormalities that drive tumor development are usually coupled with multiple alterations including the removal of methyl groups from specific amino acid residues [159,160,161]. Emerging evidence suggesting that histone demethylases (KDMs) accelerate cancer progression, metastasis, and therapy resistance has stimulated the development of specific “epipharmaceuticals”. The most advanced epipharmacochemical studies refer to lysine-specific demethylase 1 (LSD1), Figure 4. To date, several inhibitors of LSD1 were tested. LSD1 inhibitors pargyline and tranylcypromine (TCP), which also inhibit the monoaminooxydases (MAO-A and MAO-B), are successfully used in therapies for the symptomatic treatment of depression. The most specific inhibitors of LSD1, oryzon (ORY-1001) and GSK2879552, are currently in phase I and II of clinical trials against cancer. ORY-1001 is tested for treatment of relapsed or refractory acute leukemia (EudraCT Number: 2013-002447-29), whereas GSK2879552 is tested for the treatment of acute myeloid leukemia (NCT02177812) and small cell lung carcinoma (NCT02034123). Additionally, GSK2879552 is also being examined alone and in combination with the DNA methyltransferase inhibitor azacitidine in subjects with high risk myelodysplastic syndrome (NCT02929498). Clinical assessment has been also been initiated for recently synthesized compounds that promisingly passed in vivo and in vitro tests: INCB059872 in subjects with advanced malignancies (NCT02712905), INCB057643 in relapsed or refractory Ewing sarcoma (NCT03514407, NCT02842827), IMG-7289 in patients with myelofibrosis (NCT03136185), IMG-7289 with and without all-trans retinoic acid (ATRA), in patients with advanced myeloid malignancies (as acute promyelocytic leukemia is highly curable with ATRA, whereas recent data suggest that LSD1 may contribute in resistance to trans-retinoic acid [162]); similar studies are conducted in a combination with trancypromine (TCP+ATRA; NCT02261779, NCT02273102) and CC-90011 in subjects with relapsed and/or refractory solid tumors and non-Hodgkin’s lymphomas (NCT02875223) [73]).

Also, demethylases belonging to JmjC family, comprising about 20 human enzymes which are grouped into five subfamilies (KDM2/7, KDM3, KDM4, KDM5, and KDM6), are on the target list of oncopharmacology due to their contribution to tumorgenesis and cancer development. A few α-ketoglutarate analogues and Fe (II) chelators (e.g., hydoxamate, pirydinyl carboxylates), the cofactors in demethylation reactions catalyzed by JmjCs, have been reported as inhibitors of JmjC KDMs showing broad specificity and μM IC_50_. Nevertheless, new generation of more selective Jumonji enzyme inhibitors have been reported, including GSK-J1, GSKJ4 and JIB-04. It was found that JIB-04, that appears to chelate iron in the catalytic site of Jumonji enzymes and to disrupt histone substrate binding in vivo*,* lowers histone demethylase activity in tumors, reduces tumor burden and prolongs cancer survival in mice. This pan-selective inhibitor of Jumonji demethylases exhibits some selectivity in vitro for H3K4me3 demethylases and specific H3K9me3 demethylases over the H3K27 demethylases or mixed H3K9/H3K36 demethylases, but does not affect the activity of other histone-modifying enzymes [163]. GSK-J1 and its cell-active ethyl ester prodrug GSK-J4, inhibits KDM6 subfamily (KDM6A and KDM6B) [164,165]. The inhibitors exploit the H3K27me3-specific JMJ subfamily enzymes active site plasticity. As these small-molecule probes contain a propanoic acid side-chain that mimics 2-oxoglutarate side-chain binding, they are competitive with α-ketoglutarate but non-competitive with the peptide substrate. Chelation of the enzyme active site Fe (II) by GSJ-J1/J4 induces a movement of the active site ferrous ion, and at the cellular level reduces lipopolysaccharide-induced proinflammatory cytokine production in human primary macrophages, a process that depends on both JMJD3 and UTX [164].

## 6. Conclusions

In these last decades, an extremely large body of scientific literature investigating the methylation status of the epigenome of cancer cells has led to the widely accepted idea that disturbance of the balance between the methylation–demethylation process, both on the DNA and on histones, provide several potential pharmacological targets, as discussed in our review. Yet, whether to target a methylated or demethylated epigenetic state in tumors (either at the DNA or histone level) is still a double-edged sword and a careful characterization of the molecular alterations of a particular tumor type is required. The DNA methylation profile of cancer cells is, in general, characterized by global genomic hypomethylation accompanied by specific hypermethylation on tumor suppressor genes, resulting in the promotion of metastasis and poor clinical outcomes. In this case, administration of DNMTis may prove beneficial to re-express silenced tumor suppressor genes. On the one hand, SAM supplementation may be a treatment option on tumors characterized by oncogene overexpression, as this may promote the suppression of oncogene expression and prevent tumor growth. Similarly, dozens of clinical trials using HMT/PRMT inhibitors have been initiated, as discussed in Section 5, but it should not be excluded that in certain tumors suppression of EZH2 activity, mediated by PKB phosphorylation on EZH2 Serine 21, can contribute to oncogenesis [166]. Also, as DNA methylation and histone methylation are general epigenetic mechanisms, the use of drugs directed to epigenetic regulators to inhibit tumor growth will need not to be detrimental to non-cancerous cells. Overall, the picture that is emerging is that targeting epigenetic alterations either on cancer cells or on the associated vasculature will likely provide in the near future new first line or adjuvant therapeutic approaches towards several types of cancer.

## Figures and Tables

**Table 1:** Aberrant DNA methylation profiles occurring in cancer.

DNA Modification	Genetic Action	Biological Effects	References
**Hypermethylation of DNA**	Hypermethylation of promoter CpG islands	Silencing of tumor suppressor genes; Inhibition of transcription factors; Inactivation of metasasis inhibitors	[13,14,15]
	CpG shore methylation	Abnormal transcriptional inactivation	[16,17]
**Hypomethylation of DNA**	Decreased methylation in gene promoter regions	Activation of metastasis and tumor promoting genes	[18]
	Hypomethylation of gene bodies	Altered and incorrect gene expression due to activation of alternative transcription start sites (TSSs) regulatory sequences	[19]
	Global hypomethylation of genome	Chromosomal instability and reactivation of repetitive genomic sequences	[20]
	Loss of imprinting	Activation of imprinted genes (IGF-2, H19)	[21,22]

**Table 2:** Approved methyl-epigenetic drugs in oncology [76,77].

Drug Name	Epigenetic Action	Leading Center	Approval Date	Clinically Approved Indications
Azacitidine (Vidaza^®^)	DNMTs inhibiton	Celgene	May 2004	Acute Myelogenous Leukemia, AML Chronic Myelogenous Leukemia, CML Myelodysplastic Syndromes, MDS
Decitabine (Dacogen^®^)	DNMTs inhibiton	Astex Pharmaceuticals (Otsuka)	May 2006	Acute Myelogenous Leukemia, AML Chronic Myelogenous Leukemia, CML Myelodysplastic Syndromes, MDS

**Table 3:** Targeting tumor angiogenesis via DNA hypomethylating agents.

Class	Epidrug	Epigenetic Target	Molecular Target	Biological Effects of Treatment	References
Nucleoside analogs	5-azacytidine (Aza)	DNMT1	Decreased level of VEGFs: pro-angiogenic (121a, 165a) and anti-angiogenic (121b, 165b); Increased expression of TSP1, TIMP3 and CDH1 and anti-angiogenic VEGF(189b)	Decreased ECs proliferation; Decreased tumor vessel development in vivo	[34,81,82]
	Decitabine (DAC)	DNMT1	Increased expression of: EGFL7, JUNB, IGFBP3, miR126, TSP1, WIF	Decreased ECs proliferation; Decreased tumor vessel development in vivo	[80]
	Guadecitabine (SGI-110; antimetabolite of DAC)	DNMT1	Increased expression of: CDKN2A, DLEC1, RUNX3	Decreased microvessel density in vivo	[83,84]
	Zebularine (Zeb)	DNMT1	Increased level of: ICAM1, TSP1, JUNB, IGFBP3	Increased leukocyte adhesion to ECs	[33,85]
Antisense oligonucleotides	MG98	DNMT1	Re-expression of p16	Decreased cell proliferation	[86,87]
Low molecular weight molecules	RG108	DNMT1	Re-expression of p16, SRFP1, TIMP-3	Decreased cell proliferation	[88]
	Procainamide	DNMT1	Inhibition of NF-κB	Decreased cell proliferation, capillary network formation	[89,90]
	Disulfiram	DNMT1	Increased expression of: RECK	Decreased activity of MMP2 and MMP9	[91]
	Hydralazine (HYD)	DNMT1 DNMT3a DNMT3b	Re-expression of: p16, RAR-β	Decreased ability of ECs for: tube network formation, migration and proliferation; Decreased level of VEGF and microvessel density in vivo	[92]
Natural compounds (Epi-nutrients)	Curcumin	DNMT1	Decreased expression of: STAT3	Decreased ECs proliferation	[93,94]
	(−)-Epigallo-catechin-3-gallate (EGCG)	DNMT1	Increased expression of: RECK. Inhibition the activation of: HIF-α, NF-κB and VEGF expression	Decreased ability of ECs for capillary network formation; Decreased microcapillary density in vivo	[95,96,97]
	Psammaplin A (PsA)	DNMT1 HDACs		Suppression of invasion and tube formation of ECs	[98]
